# ProtoSurv: prototype-guided adaptation of computed tomography foundation model for lung-cancer prognosis prediction

**DOI:** 10.1186/s42492-026-00233-1

**Published:** 2026-09-22

**Authors:** Chengcai Liu, Shitian Li, Haolin Sang, Xingyu Huang, Chenghao Wang, Yifei Shu, Yi Wu, Jie Tian, Huimao Zhang, Lei Zhang, Shuo Wang

**Affiliations:** 1 Beijing Advanced Innovation Center for Big Data-Based Precision Medicine, School of Engineering Medicine, Beihang University, Beijing, 100191 China; 2 CAS Key Laboratory of Molecular Imaging, Beijing Key Laboratory of Molecular Imaging, Institute of Automation, Chinese Academy of Sciences, Beijing, 100190 China; 3 Department of Radiology, First Affiliated Hospital of Jilin University, Changchun, Sichuang 130021 China

**Keywords:** Lung cancer, Prognosis, Computed tomography, Vision-language model, Prototype

## Abstract

Prognosis prediction is important for precision treatment in lung cancer. Chest computed tomography (CT) and deep learning are feasible approaches for learning imaging-based prognostic representations. However, prognostic labels are difficult to obtain because survival endpoints require long-term follow-up; thus, labeled data for supervised training is limited, increasing the risk of overfitting. Vision-language pretraining offers an approach to mitigate this problem by leveraging large-scale unlabeled CT data; however, most existing methods are not specifically designed to learn prognosis-related features. We propose ProtoSurv, a prototype-guided learning framework for adapting large-scale pretrained CT representations for lung-cancer prognosis prediction under limited supervision. ProtoSurv first pretrains a CT-report vision-language model using 409,261 chest CT sequences from 104,783 patients. It then uses limited labeled prognosis data to construct class prototypes in the pretrained feature space. These prototypes guide pseudolabel assignment, confidence-based filtering, and feature calibration, allowing the selection and refinement of task-relevant unlabeled samples before downstream prognosis modeling. We evaluated ProtoSurv based on two lung-cancer prognosis tasks: progression-free survival prediction in 507 patients receiving targeted therapy and overall survival prediction in 420 patients receiving (chemo-)radiotherapy. ProtoSurv achieved area under the curve/concordance index values of 0.765/0.684 and 0.822/0.727 for the first and second datasets, respectively, outperforming the conventional clinical models and representative deep learning baselines. These results suggest that the prototype-guided adaptation can improve the use of large-scale unlabeled CT data for prognosis modeling when labeled survival data are limited.

**Supplementary Information:**

The online version contains supplementary material available at https://doi.org/10.1186/s42492-026-00233-1.

## Introduction

Lung cancer remains the leading cause of cancer-related mortality worldwide, and accurate prognostic prediction is essential for guiding personalized treatment planning and improving clinical outcomes [1–5]. Targeted therapy and (chemo-)radiotherapy are the primary treatment methods for advanced lung cancer, with progression-free survival (PFS) and overall survival (OS) as key prognostic endpoints that guide treatment decisions. Computed tomography (CT) is a routinely acquired and noninvasive imaging modality that can capture tumor morphology and intratumoral heterogeneity, providing imaging information for prognosis prediction and treatment decision support [6, 7]. However, previous prognosis models primarily relied on manually extracted clinical variables or imaging features, which have a limited ability to capture spatial heterogeneity and subtle morphological patterns distributed across three-dimensional (3D) chest CT scans [8–10].

Recent advances in deep learning have improved prognosis-prediction performance by directly learning prognostic representations from medical images [11–14]. However, a key challenge remains: prognosis requires long-term follow-up; thus, prognosis labels are difficult to obtain, and a limited amount of data is available for supervised learning [15, 16]. Consequently, models are prone to overfitting when trained solely on limited prognostic datasets.

Recent advances in vision-language pretraining have shown that leveraging large-scale unlabeled data can address the issue of limited labeled data in downstream supervised-learning tasks [17–20]. Among these approaches, contrastive language-image pretraining (CLIP) has shown promise in extracting informative image representations from large-scale unlabeled data [21, 22]. However, these methods tend to learn general features or features described in the text, whereas prognosis-related information is often not explicitly captured in CT radiology reports [23–27]. Consequently, learned features may lack the specificity required for prognosis prediction.

We address the problem of overfitting caused by the limited availability of labeled data for lung-cancer prognosis modeling by proposing ProtoSurv, which leverages large-scale unlabeled CT data to mitigate this issue. Many existing methods use unsupervised pretraining to alleviate overfitting; however, they are not specifically designed to extract prognostic features relevant to survival prediction. By contrast, ProtoSurv focuses on adapting pretrained representations to better align with prognosis tasks using class prototypes within the pretrained feature space to identify prognosis-relevant samples. ProtoSurv refines general features through hierarchical pseudo-label assignment, confidence filtering, and feature calibration to become more task-specific, ultimately improving survival prediction. This study makes the following contributions:

- We construct a large-scale chest CT-report paired dataset and perform pretraining using CLIP, enabling it to learn both imaging features and report-related information from routine clinical chest CT data.
- We propose a prototype-guided adaptive strategy that leverages prognosis data to select and optimize prognosis-relevant samples from large-scale unlabeled data through hierarchical pseudolabel assignment, confidence filtering, and feature calibration.
- We conduct experiments on prognosis tasks for both targeted therapy and (chemo-)radiotherapy, and demonstrate that ProtoSurv enhances predictive discrimination and survival-risk stratification compared with conventional clinical models and other baseline approaches.

## Methods

### Overview

**Fig. 1:**
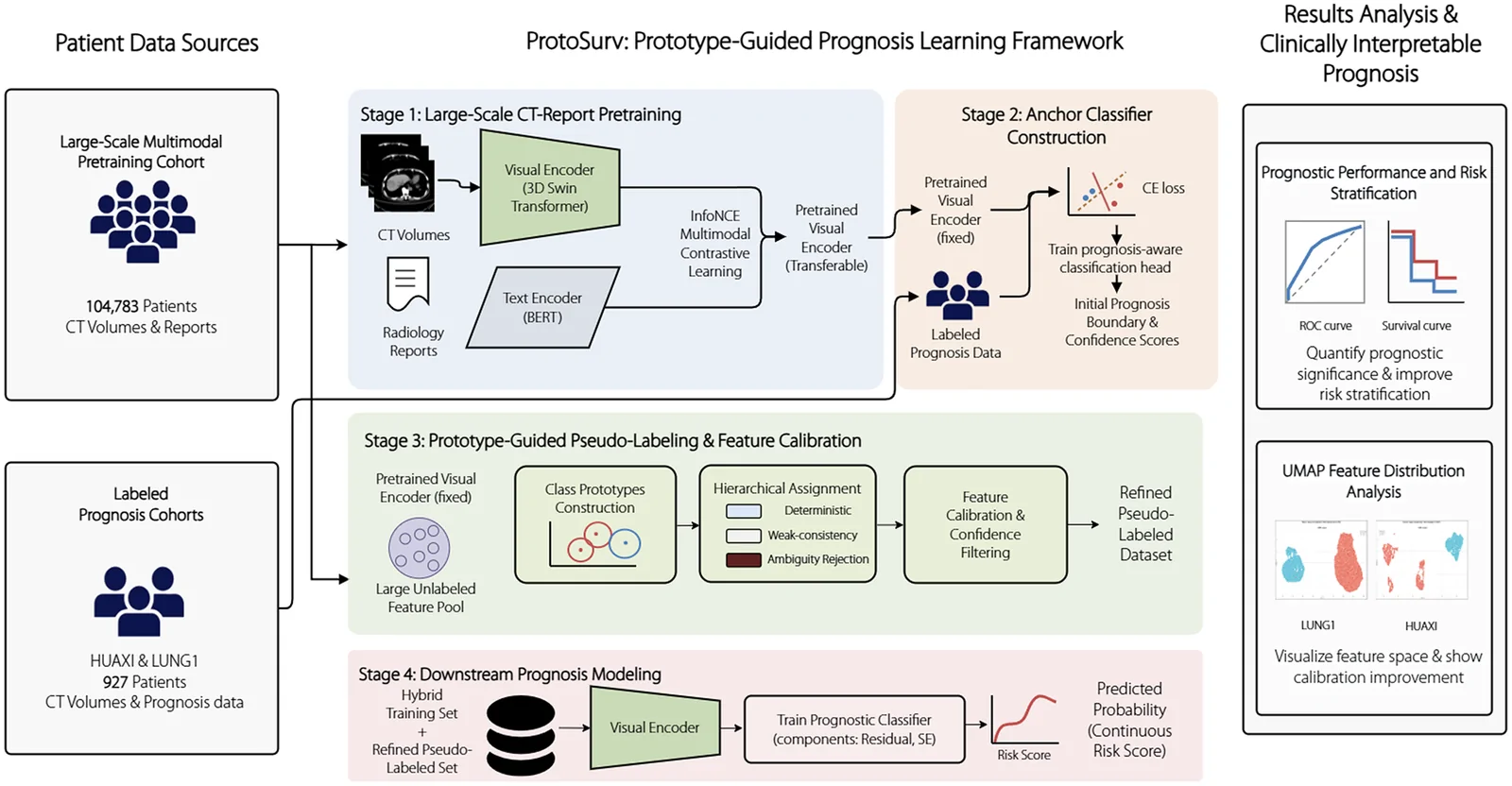
Overview of the ProtoSurv framework. The proposed framework consists of four stages. stage 1. large-scale CT-report pretraining; stage 2. anchor classifier training on labeled data; stage 3. prototype-guided pseudolabel assignment and feature calibration; stage 4. downstream prognosis modeling and evaluation

As shown in Fig. 1, ProtoSurv consists of four stages: (1) large-scale CT-report pretraining for generalizable representation learning; (2) construction of a prognosis-oriented anchor classifier on labeled survival data; (3) prototype-guided pseudolabel assignment and feature calibration on the large-scale unlabeled dataset; and (4) downstream prognosis modeling and survival risk estimation.

Specifically, we first pretrain a vision-language encoder on a large-scale CT-report dataset to learn transferable volumetric CT representations enriched with report-level radiological semantics. We then use the labeled prognosis dataset to define a task-specific class structure, where binary labels indicate whether progression (for PFS) or death (for OS) occurred before the dataset-specific median event time (i.e., mid-PFS or mid-OS, respectively). Based on these labeled samples, we train an anchor classifier and construct class prototypes in the pretrained feature space. The anchor classifier provides an initial prognosis-aware decision boundary, and the prototypes are used to identify task-relevant unlabeled samples from a large-scale dataset through hierarchical pseudolabel assignment. Candidate samples are further screened by classifier confidence to improve the pseudolabel reliability, and moderately matched samples are refined by prototype-guided feature calibration. Finally, the refined encoder is transferred to the downstream prognosis tasks. The predicted probability is used for binary discrimination and as a continuous risk score for survival analysis, and the area under the receiver operating characteristic curve (AUC), concordance index (C-index), Cox analysis, and Kaplan-Meier stratification are used for evaluation.

### Datasets

**Pretraining dataset.** For large-scale vision-language pretraining, we retrospectively collected data from 104,783 patients at the First Hospital of Jilin University between May 2023 and October 2023. The resulting dataset comprised 409,261 chest CT sequences and the corresponding radiology reports, with one report per patient. Each patient contributed at least one volumetric chest CT examination, whereas some patients had multiple CT series acquired under different imaging settings such as slice thickness and reconstruction kernel. In such cases, all CT series from the same examination were paired with the same radiology report to construct an image-text pretraining dataset. Detailed demographic characteristics and CT acquisition parameters of the pretraining cohort are summarized in Supplementary Table S1.

**Downstream prognosis dataset.** We used two downstream datasets to evaluate the transferability of the pretrained model for prognosis prediction: the HUAXI and public LUNG1 datasets.

The HUAXI dataset included 507 patients from West China Hospital who underwent targeted therapy for PFS prediction. Each patient underwent chest CT imaging at diagnosis, received a standard epidermal growth-factor receptor tyrosine kinase inhibitor treatment, and underwent at least 6 months of follow-up.

The LUNG1 [28] dataset included 420 patients and was used for OS prediction after (chemo-)radiotherapy. For both downstream datasets, pretreatment chest CT scans and the corresponding clinical outcome data were collected for prognosis modeling.

Both downstream datasets were randomly divided into training and testing sets in an 8:2 ratio. For each dataset, the 20% test set was held out for the final evaluation only, whereas all model development, including anchor-classifier training, prototype construction, threshold determination, pseudolabeling, and hyperparameter selection, was conducted exclusively on the training set. The details of the dataset characteristics and data splits are provided in Supplementary Table S2. The study was approved by the institutional review boards of West China Hospital and the First Hospital of Jilin University. The requirement for informed consent was waived.

### Large-scale vision-language pretraining

The visual encoder adopts a 3D Swin Transformer [29] backbone pretrained on the Kinetics-400 [30] dataset to capture the 3D spatial context of the chest CT volumes. Prior to the model input, the lung regions are automatically segmented using the U-Net model implemented in the lungmask library [31], and the cropped lung-focused CT volume is used as the visual input. Given a CT volume *x*, the visual encoder $E_v(\cdot)$ maps it to a compact visual representation:

1 $$\mathbf{v} = E_v(x), \quad \mathbf{v} \in \mathbb{R}^{d}$$

This design allows the model to focus on lung-related pathological and anatomical structures, while suppressing irrelevant background information.

The text encoder is implemented using a pretrained Bidirectional Encoder Representations from Transformers [32] model to extract semantic representations from the radiology reports. Given a report text *r*, the encoder $E_t(\cdot)$ produces a textual embedding, which is further projected into the same embedding space as the visual features:

2 $$\mathbf{t} = E_t(r), \quad \mathbf{t} \in \mathbb{R}^{d}$$

By aligning visual and textual embeddings, the model learns CT representations enriched with report-level radiological semantics.

For a mini-batch containing *N* matched CT-report pairs, positive pairs are formed by the CT scan and report from the same patient, whereas all remaining cross-patient pairs within the batch are treated as negative pairs. We adopt symmetric InfoNCE loss to align visual and textual representations:

3 $$\begin{aligned}\mathcal{L}_{\mathrm{InfoNCE}} = -\frac{1}{2N} \sum_{i=1}^{N} \Biggl[ \log \frac{\exp(\mathbf{v}_i^\top \mathbf{t}_i / \tau)}{\sum_{j=1}^{N} \exp(\mathbf{v}_i^\top \mathbf{t}_j / \tau)} \\+ \log \frac{\exp(\mathbf{t}_i^\top \mathbf{v}_i / \tau)}{\sum_{j=1}^{N} \exp(\mathbf{t}_i^\top \mathbf{v}_j / \tau)} \Biggr]\end{aligned}$$

where *τ* is a learnable temperature parameter and both $\mathbf{v}_i$ and $\mathbf{t}_i$ are L2-normalized. Through this multimodal contrastive objective, the visual encoder learns CT representations aligned with the report-level semantic information.

After pretraining, the text encoder is discarded, and the pretrained visual encoder is transferred to the downstream prognosis tasks.

### Prognosis-oriented anchor classifier

The pretrained visual encoder captures general radiological semantics; however, its representation is not directly optimized for prognosis prediction. To bridge this gap, we construct a prognosis-oriented anchor classifier using a labeled dataset with survival follow-up. This classifier has two main purposes: (1) providing an initial prognosis-aware decision boundary in the pretrained feature space; and (2) estimating the confidence scores for unlabeled samples during pseudolabel screening.

Let $\mathcal{D}_L = \{(x_i, y_i)\}_{i=1}^{M}$ denote the labeled prognosis dataset, where *M* is the number of patients and $y_i \in \{0,1\}$ indicates the binary prognosis status such as the mid-PFS progression status or mid-OS survival status. For each sample $x_i$, the pretrained visual encoder extracts a feature vector:

4 $$f_i = E(x_i)$$

where *E(·)* denotes the pretrained visual encoder. A trainable fully connected classification head *g(·)* is then attached to the encoder to produce the class probabilities:

5 $$\mathbf{p}_i = g(f_i), \quad \mathbf{p}_i = [p_{i,0}, p_{i,1}]$$

where $p_{i,c}$ represents the probability that the sample $x_i$ belongs to class *c*. The anchor classifier is trained on $\mathcal{D}_L$ using the binary cross-entropy loss:

6 $$\mathcal{L}_{\mathrm{anchor}} = - \frac{1}{M}\sum_{i=1}^{M}\sum_{c=0}^{1} y_{i,c}\log p_{i,c}$$

The resulting anchor classifier provides a prognosis-aware confidence estimate and acts as an auxiliary semantic filter for the subsequent pseudolabel assignment.

### Prototype-guided pseudo-label assignment and feature calibration

**Fig. 2:**
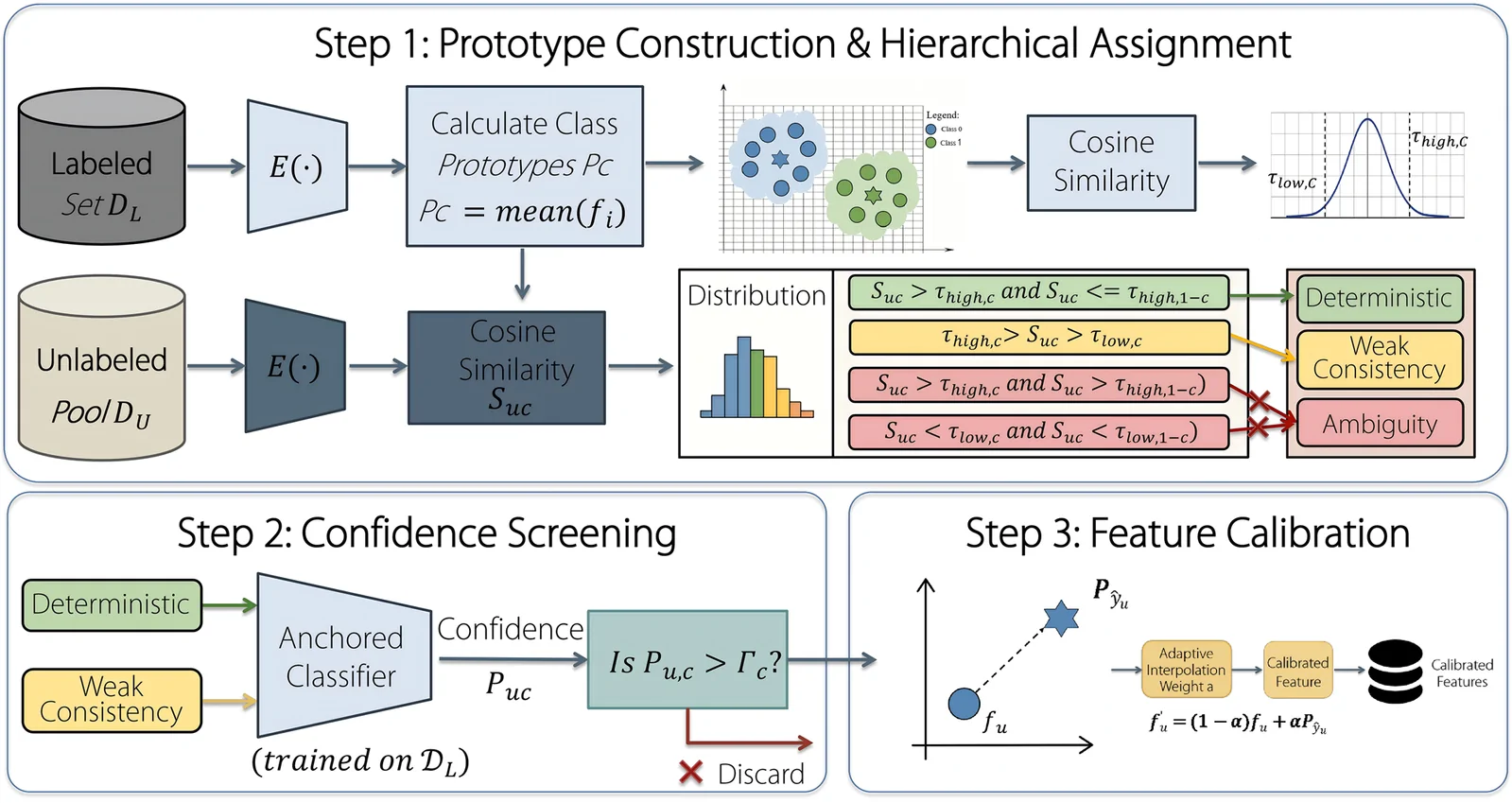
Prototype-guided pseudolabel assignment and feature calibration in ProtoSurv. The proposed pseudolabel assignment and feature-calibration mechanism consist of three modules. step 1. prototype-based hierarchical pseudolabel assignment (deterministic assignment, weak-consistency assignment, ambiguity rejection); step 2. anchor classifier-confidence filtering; step 3. prototype-guided adaptive feature calibration

We propose a prototype-guided strategy that combines prototype similarity, hierarchical pseudolabel assignment, confidence screening, and feature calibration to further exploit the representation potential of a large-scale unlabeled dataset, as shown in Fig. 2.

#### Class prototype construction

Based on the labeled prognosis dataset $\mathcal{D}_L$, we construct class prototypes in the pretrained feature space. For each class $c \in \{0,1\}$, the prototype $\mathbf{P}_c$ is defined as the centroid of all labeled samples belonging to that class:

7 $$\mathbf{P}_c = \frac{1}{|\mathcal{D}_L^c|}\sum_{x_i \in \mathcal{D}_L^c} f_i$$

where $f_i = E(x_i)$ and $\mathcal{D}_L^c$ denote a subset of labeled samples with class label *c*. These prototypes summarize the global semantic centers of the prognosis-related patterns in the feature space.

For each unlabeled sample $x_u$ from the large-scale dataset $\mathcal{D}_U$, feature representation is extracted as follows:

8 $$f_u = E(x_u)$$

We then compute the cosine similarity of each prototype as follows:

9 $$s_{u,c} = \cos(f_u, \mathbf{P}_c)$$

We define the adaptive decision boundaries for each class by calculating the similarity distribution of labeled samples to their own class prototype and using the upper quartile $Q_3$ and lower quartile $Q_1$ as the high- and low-confidence thresholds, denoted by $\tau_{\mathrm{high},c}$ and $\tau_{\mathrm{low},c}$, respectively.

#### Hierarchical pseudolabel assignment

Pseudolabel assignment is divided into three levels based on the relative position of an unlabeled sample in the prototype space.

**(1) Deterministic assignment.** If an unlabeled sample is highly similar to a one-class prototype and not highly similar to another, that is,

10 $$s_{u,c} > \tau_{\mathrm{high},c}, \quad s_{u,1-c} \leq \tau_{\mathrm{high},1-c}$$

a hard pseudolabel is assigned as follows:

11 $$\hat{y}_u = c$$

** (2) Weak-consistency assignment.** If the similarity lies between $\tau_{\mathrm{low},c}$ and $\tau_{\mathrm{high},c}$, the sample is assigned to the class with the highest similarity score and is marked as a weak-consistency sample.

**(3) Ambiguity rejection.** Samples simultaneously satisfying both high thresholds or failing to reach either low threshold are regarded as ambiguous or outlier samples and are excluded from the current iteration.

This hierarchical design prevents overly aggressive pseudolabel expansion, while retaining moderately informative unlabeled samples for further calibration.

#### Confidence-based filtering

Prototype similarity alone may still introduce noisy pseudolabels owing to feature overlap and domain heterogeneity. Therefore, after the initial pseudolabel assignment, we use an anchor classifier to estimate the confidence for each candidate unlabeled sample. For an unlabeled sample $x_u$, the anchor classifier outputs the following:

12 $$\mathbf{p}_u = [p_{u,0}, p_{u,1}]$$

Only samples that satisfy the class-specific confidence threshold are retained:

13 $$\mathbf{p}_{u,\hat{y}_u} > \Gamma_{\hat{y}_u}$$

where $\Gamma_c$ is the confidence threshold for class *c*, which is selected based on the validation performance. This step filters out uncertain samples from a probability perspective and improves the reliability of the pseudo-labeled set.

#### Prototype-guided feature calibration

For weak-consistency samples, using the original features directly may lead to unstable supervision because these samples lie near the class boundary. We address this issue by introducing a prototype-guided feature-calibration strategy that adaptively pulls the features of a pseudolabeled sample toward its assigned class prototype.

For a weak-consistency sample $x_u$ with a pseudolabel $\hat{y}_u$, the calibrated feature $f\prime_u$ is defined as follows:

14 $$f\prime_u = (1-\alpha) f_u + \alpha \mathbf{P}_{\hat{y}_u}$$

where *α* denotes an adaptive interpolation weight determined by the relative prototype similarity:

15 $$\alpha = \mathrm{clip}\left( \eta \cdot \frac{\tau_{\mathrm{high},\hat{y}_u} - s_{u,\hat{y}_u}} {\tau_{\mathrm{high},\hat{y}_u} - \tau_{\mathrm{low},\hat{y}_u}}, 0, 0.5 \right)$$

where *η* is a predefined scaling factor controlling the calibration strength. Intuitively, samples closer to the lower-confidence boundary receive a larger correction, whereas samples already close to the prototype require fewer adjustments. This operation improves the intraclass compactness and reduces the negative effects of noisy pseudolabels during downstream training.

After confidence screening and feature calibration, we obtain a refined pseudolabeled dataset $\mathcal{D}_{\mathrm{pseudo}}$, which is combined with the original labeled dataset for downstream prognosis modeling.

### Downstream prognosis modeling

#### Hybrid training-set construction

We address the scarcity of prognostic labels by constructing a hybrid augmented dataset and combining the original labeled dataset and selected pseudolabeled samples:

16 $$\mathcal{D}_{\mathrm{total}} = \mathcal{D}_{\mathrm{train}} \cup \mathcal{D}_{\mathrm{pseudo}}$$

Here, $\mathcal{D}_{\mathrm{train}}$ denotes the original clinical dataset with explicit prognosis labels and $\mathcal{D}_{\mathrm{pseudo}}$ contains high-confidence pseudolabeled samples obtained from the large-scale unlabeled dataset.

#### Prognostic classifier

Based on the pretrained image encoder, we design a dedicated prognostic classifier for binary risk prediction. The encoder is used as the feature-extraction backbone, and its output is fed into a customized prognosis head for the final classification. During downstream training, the encoder parameters are frozen to preserve the general multimodal knowledge learned from large-scale pretraining and reduce overfitting on the limited prognosis dataset.

**Fig. 3:**
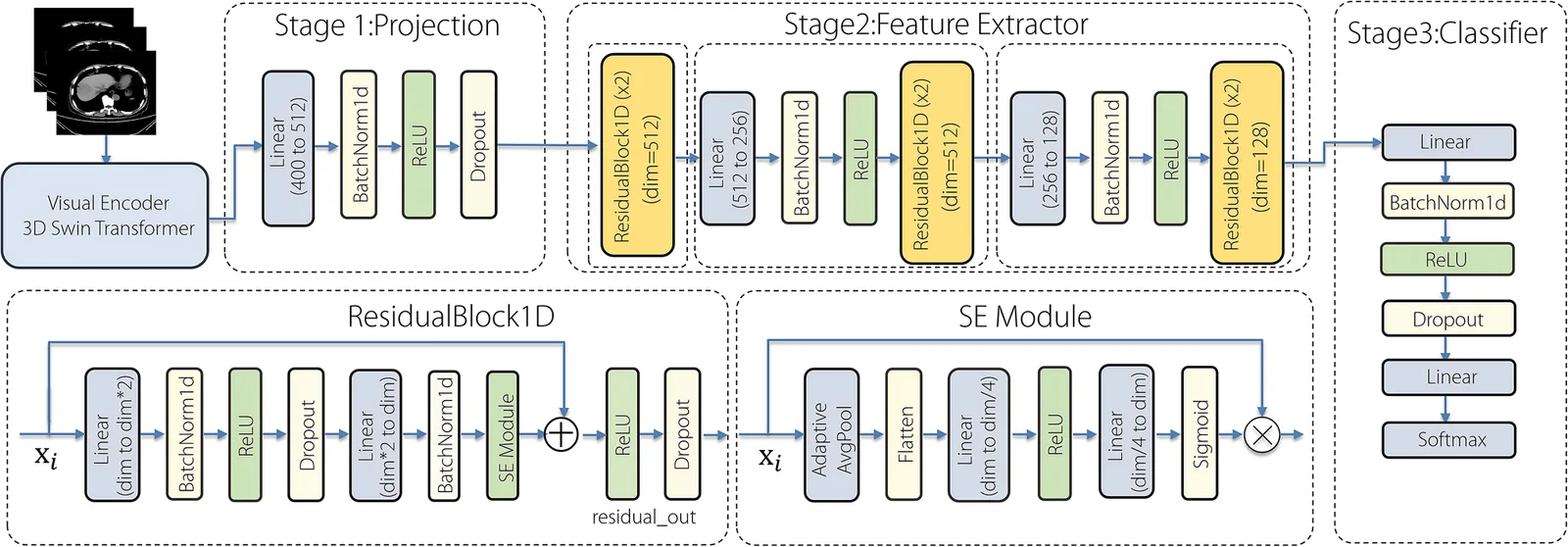
Structure of the prognostic classifier

The prognostic classifier is built on a one-dimensional residual architecture and consists primarily of the following components, as shown in Fig. 3:

- **Residual bottleneck blocks.** Multiple bottleneck-style residual blocks are employed to enhance nonlinear feature transformation while maintaining efficient gradient propagation through skip connections.
- **Squeeze-and-excitation (SE) attention.** SE modules are embedded into residual blocks to model channel-wise dependencies and adaptively reweight feature responses, thereby emphasizing prognosis-relevant patterns and suppressing redundant noise.

Given the extracted feature, *f*, the prognostic classifier outputs the predicted probability of the high-risk class:

17 $$\hat{p} = h(f)$$

where *h(·)* denotes the overall prognosis-prediction head. The model is optimized using the binary cross-entropy loss:

18 $$\mathcal{L}_{\mathrm{prog}} = - \frac{1}{|\mathcal{D}_{\mathrm{total}}|} \sum_{(x,y)\in \mathcal{D}_{\mathrm{total}}} \Bigl[y\log \hat{p} + (1-y)\log(1-\hat{p})\Bigr]$$

#### Survival-risk estimation and evaluation

Prognosis modeling is formulated as a binary classification task for each downstream dataset, where the label indicates whether progression (PFS) or death (OS) occurred before the dataset-specific median event time. The downstream task is formulated as a binary prognostic classification; however, the predicted probability $\hat{p}$ naturally reflects the individualized risk level. Therefore, we directly use $\hat{p}$ as a continuous risk score for the survival analysis, where a higher predicted probability indicates a higher risk of progression or death.

Based on the predicted risk score, we calculate the C-index to assess the ability of the model to rank patients according to their survival risk. In addition, the risk score is incorporated into a Cox proportional-hazards model to quantify its prognostic significance with respect to time-to-event outcomes. This strategy bridges binary classification and survival modeling, enabling both discrimination assessment and clinically interpretable prognostic analysis.

### Implementation details

Model training and statistical analysis are performed using Python 3.12.7 with PyTorch 2.5.1, torchvision 0.20.1, scipy 1.13.1, pandas 2.2.2, lifelines 0.30.3, and numpy 1.26.4. For the threshold-based performance evaluation, the cutoff for each prediction task is determined in the training set by maximizing the Youden index and is subsequently applied unchanged to the testing datasets. The *t*-test is used to assess intergroup differences in continuous variables, and the chi-square test is used to assess intergroup differences in categorical variables. Unless otherwise specified, the 95%CIs for the performance metrics are estimated using 1000 patient-level bootstrap resamples. All tests are two-sided, and the results are considered statistically significant at a *p*-value * < 0.05*. The C-index reported in the main experiments is a time-dependent index evaluated at the median PFS or OS time point, which is consistent with the fixed-horizon binary task formulation. In contrast, Harrell’s C-index is computed over the complete continuous follow-up period and is reported in the additional survival analysis for both ProtoSurv and the native Cox-loss model to capture the global prognostic ranking.

## Results

### Model-performance comparison and survival-risk stratification

We systematically compare the predictive performance of ProtoSurv against three baseline models (Clinical model, DenseNet [33], and M3D [34]) for both the HUAXI and LUNG1 datasets. As shown in Table 1, on the HUAXI dataset, ProtoSurv achieves an AUC of 0.765 (95%CI: 0.673–0.856) and C-index of 0.684 (95%CI: 0.622–0.755) for predicting the mid-PFS status. These results are higher than those obtained using the clinical model (AUC: 0.590, C-index: 0.576), DenseNet (AUC: 0.594, C-index: 0.568), and M3D (AUC: 0.636, C-index: 0.606). Notably, ProtoSurv achieves a recall of 0.854 (95%CI: 0.755–0.954), which is higher than the best baseline value (M3D: 0.717), indicating its superior capability in identifying high-risk patients.

**Table 1:** Performance comparison of different models for lung-cancer prognosis prediction on the HUAXI and LUNG1 datasets

Dataset	Metric	Clinical model	DenseNet	M3D	ProtoSurv
HUAXI	C-index	0.576 [0.462–0.696]	0.568 [0.482–0.657]	0.606 [0.521–0.695]	**0.684 [0.622–0.755]**
	AUC	0.590 [0.484–0.697]	0.594 [0.467–0.714]	0.636 [0.518–0.752]	**0.765 [0.673–0.856]**
	Accuracy	0.554 [0.455–0.653]	0.602 [0.494–0.699]	0.624 [0.525–0.713]	**0.663 [0.574–0.753]**
	F1-score	0.571 [0.447–0.677]	0.612 [0.480–0.719]	0.635 [0.519–0.737]	**0.707 [0.614–0.791]**
	Precision	0.526 [0.392–0.655]	0.553 [0.411–0.686]	0.569 [0.441–0.695]	**0.603 [0.487–0.719]**
	Recall	0.625 [0.489–0.756]	0.684 [0.539–0.829]	0.717 [0.585–0.845]	**0.854 [0.755–0.954]**
LUNG1	C-index	0.538 [0.460–0.625]	0.605 [0.527–0.699]	0.642 [0.553–0.741]	**0.727 [0.665–0.798]**
	AUC	0.525 [0.385–0.651]	0.641 [0.527–0.767]	0.643 [0.533–0.758]	**0.822 [0.737–0.903]**
	Accuracy	0.529 [0.414–0.643]	0.595 [0.500–0.702]	0.607 [0.512–0.703]	**0.750 [0.667–0.845]**
	F1-score	0.459 [0.295–0.605]	0.630 [0.512–0.741]	0.629 [0.500–0.731]	**0.734 [0.613–0.832]**
	Precision	0.483 [0.312–0.667]	0.547 [0.426–0.685]	0.560 [0.435–0.690]	**0.725 [0.576–0.854]**
	Recall	0.438 [0.258–0.622]	**0.744 [0.615–0.872]**	0.718 [0.568–0.848]	**0.744 [0.585–0.881]**

Values are presented as mean [95%CI]. Bold values indicate the best performance for each metric within each dataset. AUC: Area under the receiver operating characteristic curve; C-index: Concordance index

On the LUNG1 dataset, ProtoSurv also achieves a higher performance, with an AUC of 0.822 (95%CI: 0.737–0.903) and C-index of 0.727 (95%CI: 0.665–0.798) for predicting the mid-OS status. ProtoSurv achieves AUC improvements of 0.297, 0.181, and 0.179 compared with the clinical model (AUC: 0.525, C-index: 0.538), DenseNet (AUC: 0.641, C-index: 0.605), and M3D (AUC: 0.643, C-index: 0.642), respectively. Furthermore, ProtoSurv achieves a higher accuracy (0.750) and F1-score (0.734) than all baseline models.

**Fig. 4:**
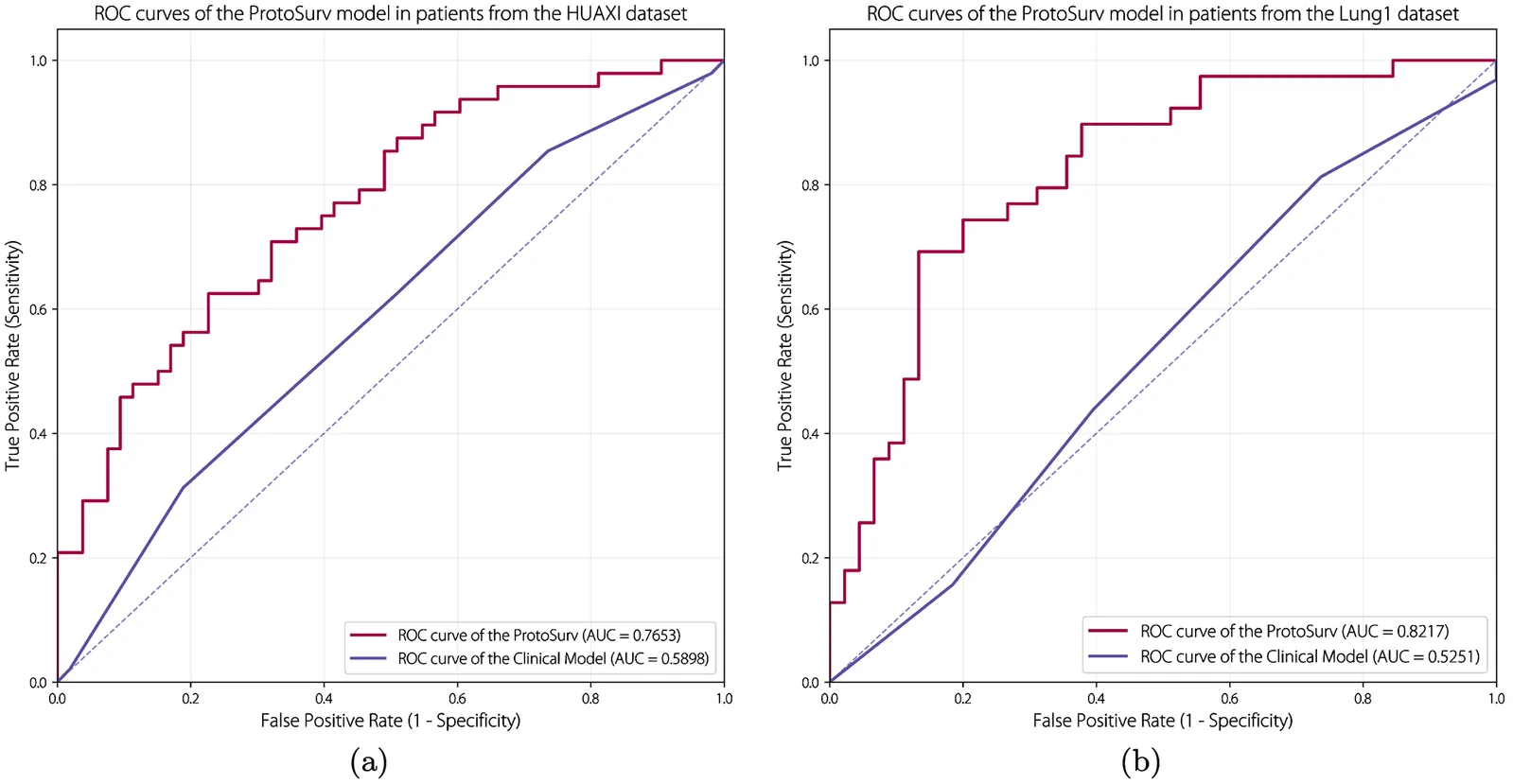
Receiver operating characteristic curves of ProtoSurv and the clinical model on the HUAXI and LUNG1 datasets. **a** ROC curves of ProtoSurv and the clinical model on the HUAXI dataset; **b** ROC curves of ProtoSurv and the clinical model on the LUNG1 dataset. ROC: Receiver operating characteristic

Figure 4 presents the receiver operating characteristic (ROC) curves comparing ProtoSurv and the clinical model on both datasets. For the HUAXI dataset (Fig. 4a), ProtoSurv achieved an AUC of 0.765, substantially higher than the value of 0.590 achieved using the clinical model. For the LUNG1 dataset (Fig. 4b), ProtoSurv achieved an AUC of 0.822, while the clinical model achieved only 0.525. These results demonstrate that ProtoSurv learns more discriminative prognostic features from imaging data than models that rely solely on limited clinical factors.

**Fig. 5:**
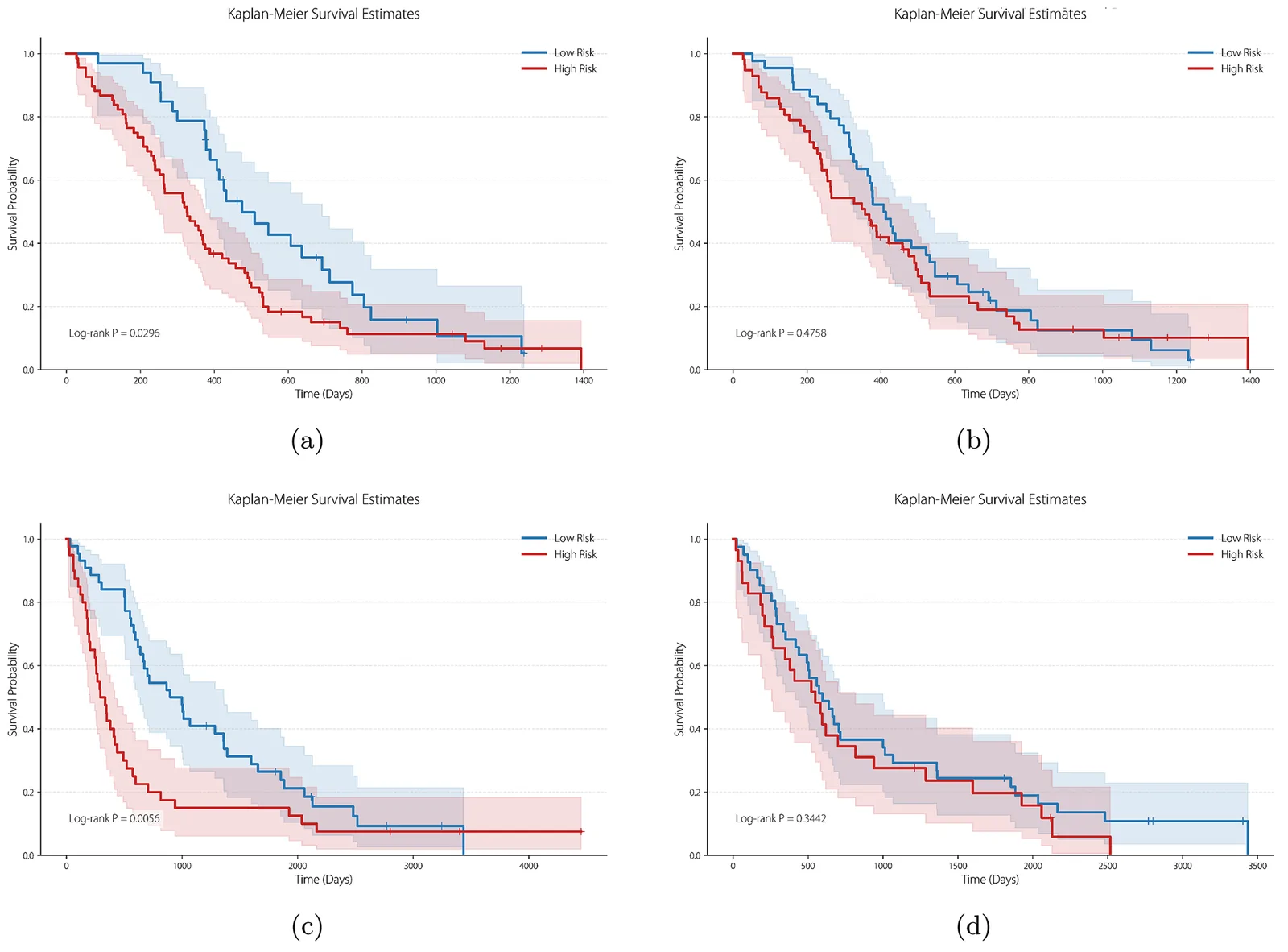
Kaplan-Meier survival curves for risk stratification on the HUAXI and LUNG1 datasets. **a** Kaplan-Meier curve of the ProtoSurv model on the HUAXI dataset; **b** Kaplan-Meier curve of the clinical model on the HUAXI dataset; **c** Kaplan-Meier curve of the ProtoSurv model on the LUNG1 dataset; **d** Kaplan-Meier curve of the clinical model on the LUNG1 dataset

We further stratified patients into high- and low-risk groups based on the predicted risk scores of ProtoSurv and performed Kaplan-Meier survival analysis. As shown in Fig. 5, on the HUAXI dataset, a significant difference in PFS was observed between high- and low-risk groups stratified by ProtoSurv (log-rank *P = 0.0296*) (Fig. 5a), whereas the clinical model-based stratification failed to produce a statistically meaningful separation (log-rank *P = 0.4758*) (Fig. 5b). Similarly, on the LUNG1 dataset, ProtoSurv stratified patients into high- and low-risk groups with significantly different OS values (Fig. 4c), while the clinical model showed limited stratification efficacy (Fig. 4d). In addition, decision-curve analysis showed that ProtoSurv achieved a higher net benefit across a threshold probability range of 0.3–0.6 compared with the Treat All and Treat None baseline strategies (Supplementary Fig. S1), indicating its potential clinical utility. These results suggest that ProtoSurv provides better survival-risk stratification than the clinical model for these datasets.

We further compared ProtoSurv with a supervised transfer, confidence-based pseudolabeling, and FixMatch to assess whether conventional semisupervised learning strategies may provide comparable benefits from the unlabeled feature pool. The detailed results are provided in Supplementary Table S3.

Compared with the supervised transfer and conventional semisupervised learning baselines, ProtoSurv achieved the highest AUC, F1-score, and recall for both HUAXI and LUNG1, demonstrating consistent discriminative performance across the different evaluation metrics. These results suggest that prototype-guided pseudolabel assignment and feature calibration enable a more effective utilization of unlabeled CT data than conventional confidence-based pseudolabeling and consistency-based semisupervised learning strategies.

### Feature-space visualization analysis

We examine the effect of our proposed prototype-guided pseudolabel assignment and feature calibration mechanism on the feature space by extracting the features of all samples from the large-scale pretraining dataset and comparing the feature distributions obtained by (1) hard assignment based solely on cosine similarity to the two class prototypes and (2) our complete mechanism. Specifically, before calibration, unlabeled samples are simply assigned to the prototype class with the closer cosine distance, without any filtering or feature optimization. After calibration, the complete pipeline for the proposed hierarchical pseudolabel assignment, confidence filtering, and prototype-guided feature calibration is applied.

**Fig. 6:**
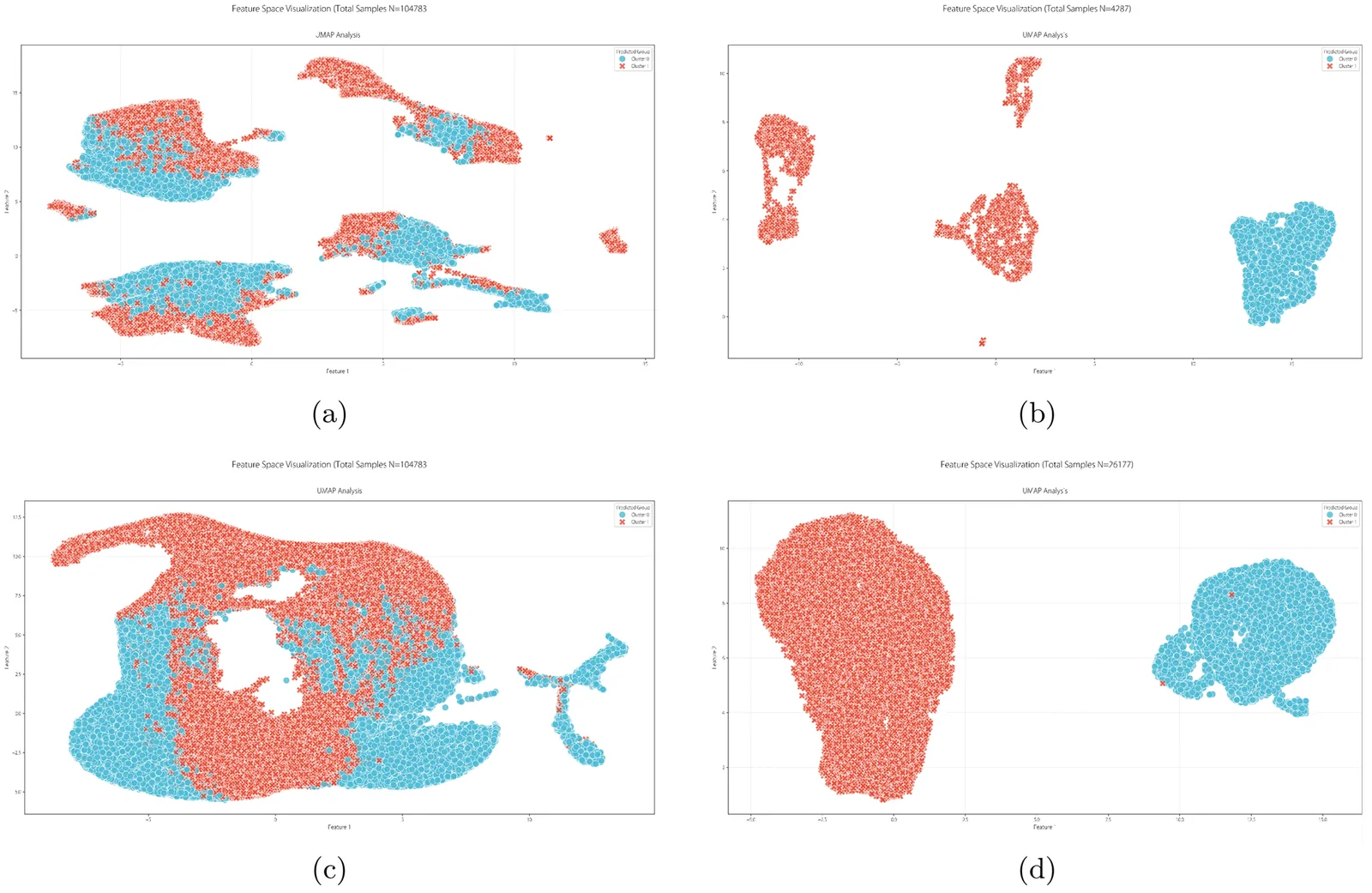
Uniform manifold approximation and projection visualization of features from the large-scale pretraining dataset before and after applying our method. Class prototypes are constructed using labeled samples from the HUAXI and LUNG1 datasets, and the visualized feature points are sampled from the pretraining dataset: **a** before our method (prototypes from HUAXI); **b** after our method (prototypes from HUAXI); **c** before our method (prototypes from LUNG1); **d** after our method (prototypes from LUNG1)

As shown in Fig. 6, when only cosine distance-based hard assignment is used (Fig. 6a, 6c), the features of different classes exhibit significant mixing and overlap, with blurred interclass boundaries and loose intraclass distributions. After applying the proposed hierarchical pseudolabel assignment, confidence filtering, and prototype-guided feature-calibration methods (Fig. 6b, 6d), the feature distributions of the same class become tighter, interclass separability is improved, and clearer decision boundaries are formed.

For quantitative feature-space evaluation, the high-dimensional features are first reduced to 50 dimensions using principal component analysis, and the quantitative metrics are calculated in this reduced feature space. Uniform manifold approximation and projection (UMAP) was used only for visualization. The quantitative feature-space evaluation supported the UMAP findings. In HUAXI, the mean within-class distance decreases from 22.8048 to 20.1531, and the interclass centroid distance increases from 17.9930 to 62.2107; accordingly, the separation-to-compactness ratio increases from 0.7890 to 3.0869, and the silhouette score increases from 0.1182 to 0.5875. Similar trends are observed in LUNG1, where the mean within-class distance decreases from 22.5766 to 16.1513, the interclass distance increases from 15.1069 to 26.6441, the separation-to-compactness ratio increases from 0.6691 to 1.6497, and the silhouette score increases from 0.1080 to 0.3310. These results indicate improved feature compactness and class separation after the complete pipeline, reflecting the combined effects of sample selection, confidence filtering, and prototype-guided feature calibration.

### Ablation study

We conduct an incremental ablation study using the ProtoSurv framework to validate the effectiveness of each proposed component (augmentation and feature calibration), as presented in Table 2.

**Table 2:** Ablation analysis of data augmentation and feature calibration in the ProtoSurv framework

Dataset	Metric	W/O Aug.	Aug. w/o Pull	Ours (ProtoSurv)
HUAXI	C-index	0.649 [0.577–0.726]	0.665 [0.596–0.741]	**0.684 [0.622–0.755]**
	AUC	0.715 [0.611–0.817]	0.738 [0.633–0.935]	**0.765 [0.673–0.856]**
	Accuracy	0.614 [0.515–0.703]	**0.683 [0.594–0.772]**	0.663 [0.574–0.753]
	F1-score	0.621 [0.509–0.717]	0.667 [0.552–0.771]	**0.707 [0.614–0.791]**
LUNG1	C-index	0.689 [0.621–0.767]	0.715 [0.650–0.788]	**0.727 [0.665–0.798]**
	AUC	0.768 [0.673–0.867]	0.804 [0.714–0.888]	**0.822 [0.737–0.903]**
	Accuracy	0.714 [0.619–0.810]	**0.762 [0.667–0.845]**	0.750 [0.667–0.845]
	F1-score	0.721 [0.600–0.822]	0.722 [0.582–0.829]	**0.734 [0.613–0.832]**

Values are presented as mean [95%CI]. Bold values indicate the best performance for each metric within each dataset. AUC: Area under the receiver operating characteristic curve; C-index: concordance index

**Impact of augmentation.** Adding data augmentation (comparing W/O Aug. with Aug. w/o Pull) improves the model performance across most metrics for both the HUAXI and LUNG1 datasets. On the HUAXI dataset, augmentation increases the C-index from 0.649 [0.577–0.726] to 0.665 [0.596–0.741] and the AUC from 0.715 [0.611–0.817] to 0.738 [0.633–0.935], demonstrating its ability to enhance model generalization. Similarly, on the LUNG1 dataset, the C-index increases from 0.689 [0.621–0.767] to 0.715 [0.650–0.788], and the AUC increases from 0.768 [0.673–0.867] to 0.804 [0.714–0.888], suggesting a similar effect of augmentation across the two datasets. However, the lack of feature calibration limits the optimization of the feature-space structure, which as teflected in the UMAP visualization is evident in the feature UMAP visualization results. When only augmentation is applied (without feature calibration), the feature distributions of the different classes still exhibit partial mixing and overlapping. This limitation restricts the ability of the model to capture fine-grained feature differences, resulting in inconsistent performance improvements across metrics.

**Necessity of feature calibration.** Augmentation expands the data distribution and improves generalization; however, integrating feature calibration (forming our complete ProtoSurv framework) further calibrates the feature space and boosts model performance, which is supported by Table 2, Supplementary Table S4, and the feature UMAP results. On the HUAXI dataset, our ProtoSurv framework achieves the best performance in key metrics: C-index reaches 0.684 [0.622–0.755], AUC increases to 0.765 [0.673–0.856], and the F1-score increases to 0.707 [0.614–0.791], which is a notable improvement compared to Aug. w/o Pull. On the LUNG1 dataset, our method also outperforms the other two ablated versions in core metrics, with a C-index of 0.727 [0.665–0.798] and AUC of 0.822 [0.737–0.903], confirming the necessity of Feature calibration. Correspondingly, feature UMAP visualization reflects the effect of Feature calibration on the feature space. After integrating feature calibration into the augmentation pipeline, the feature distributions of the same class become tighter, the interclass separation becomes clearer, and the decision boundaries are better defined, reducing the overlap observed in the Aug. w/o Pull group. This indicates that Feature calibration complements the limitations of single Augmentation, realizing the dual optimization of data-distribution expansion and feature-space calibration. By guiding feature aggregation and separation, feature calibration enables the model to better capture discriminative features related to survival prediction, leading to a more consistent performance across the two datasets.

These results demonstrate that our proposed prototype-guided mechanism can leverage latent semantic information in large-scale pretraining data. Hierarchical pseudolabel assignment, confidence filtering, and adaptive feature calibration are used to alleviate the feature-mixing problem caused by pure cosine-distance-based hard assignments, improve the discriminability and compactness of the feature space, and provide higher-quality feature representations for downstream prognosis modeling.

### Model-interpretability analysis

**Fig. 7:**
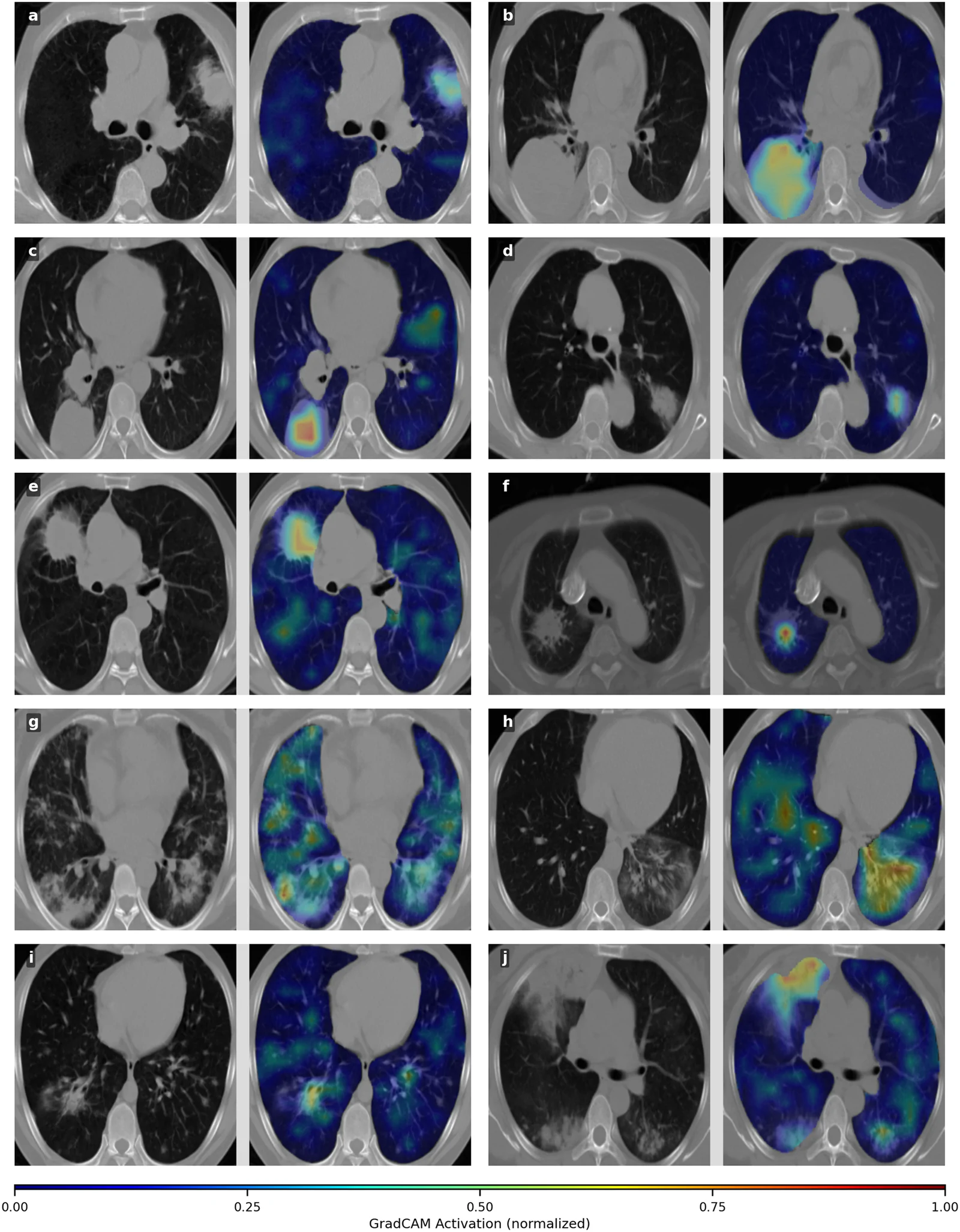
Gradient-weighted class activation mapping visualizations of representative prognosis predictions. For each representative case **a–j**, the original axial computed tomography image is shown on the left and the corresponding gradient-weighted class activation mapping overlay is shown on the right. The activation maps are normalized to the range of 0–1, with warmer colors indicating image regions that contributed more strongly to the model prediction

We generated gradient-weighted class activation mapping activation maps for representative patients from the HUAXI and LUNG1 datasets to qualitatively investigate the imaging patterns that contribute to prognosis prediction. As shown in Fig. 7, regions with relatively high activation frequently overlap with dominant pulmonary lesions and their surrounding parenchymal abnormalities. In several cases, activation was concentrated within or adjacent to the primary lesion, whereas other cases showed more spatially distributed attention involving bilateral or multifocal pulmonary abnormalities.

### Additional survival analysis

We perform additional Cox regression analysis and compare ProtoSurv with the native Cox-loss model to further evaluate whether the binary prognosis formulation retained continuous-time survival information. The native Cox-loss model uses the same pretrained encoder but directly optimizes the labeled prognosis datasets with Cox partial likelihood loss using complete survival times and censoring indicators, without prototype-guided pseudolabel assignment, confidence filtering, or feature calibration. Detailed implementation information is provided in the Supplementary Methods.

The continuous ProtoSurv risk score is significantly associated with PFS for HUAXI (HR, 1.61; 95%CI: 1.01–2.57; *P=0.0467*) and OS for LUNG1 (HR, 2.20; 95%CI: 1.34–3.61; *P=0.0018).* ProtoSurv achieves higher Harrell’s C-indices than the native Cox-loss model for both HUAXI (0.638 vs 0.600) and LUNG1 (0.680 vs 0.652), indicating that the binary prognosis formulation retained meaningful prognostic discrimination. The detailed results are provided in Supplementary Table S5.

## Discussion

This study addresses an important limitation of CT-based lung-cancer prognosis modeling: large-scale pretraining can provide transferable representations; however, these representations are not necessarily aligned with survival-related targets. ProtoSurv is designed to bridge this gap by using limited prognosis data to guide the selection and refinement of unlabeled samples. Rather than simply enlarging the training set, the framework introduces a prognosis-oriented structure into the pretrained feature space through prototype-guided pseudolabel assignment, confidence filtering, and feature calibration.

These findings suggest that prognostic adaptation may require more than a generic representation transfer. CT reports mainly describe visible anatomical and pathological findings, whereas prognosis is influenced by treatment response, tumor biology, and subtle imaging patterns that may not be explicitly recorded in the reports. Therefore, directly transferring a vision-language pretrained encoder may retain useful general semantics but still lacks sufficient prognosis specificity. By constructing class prototypes from labeled prognosis data, ProtoSurv uses limited outcome labels to identify prognosis-relevant unlabeled samples and reduce noisy pseudolabels. The feature-calibration step further refines weakly matched samples, which helps reduce feature ambiguity and encourages a more discriminative prognosis-oriented representation space.

This design is particularly relevant for medical-prognosis tasks, where labeled survival data are difficult to collect but unlabeled imaging archives are abundant. This framework suggests a practical method to extract useful supervision information from routine CT data without requiring additional manual follow-ups for prognosis. More broadly, this study indicates that the clinical value of medical pretraining may depend on the data scale as well as whether pretrained representations can be reshaped toward clinically meaningful endpoints.

This study has several limitations. First, this study is retrospective, and a larger multicenter validation is needed to assess robustness across institutions and imaging protocols. Second, the prognosis is formulated as a median-time binary-classification task, which does not fully exploit the temporal structure of survival data. Future work may integrate prototype-guided adaptation with survival-specific objectives and further explore the biological significance of the learned prognostic features. In addition, a systematic comparison between CT-report vision-language pretraining and purely visual self-supervised pretraining under identical conditions remains an important future direction.

## Conclusions

ProtoSurv provides a prototype-guided strategy for adapting large-scale CT pretraining to label-scarce lung-cancer prognosis prediction. By selecting and refining prognosis-relevant unlabeled samples, the framework surpasses direct feature transfer and reshapes generic pretrained representations into a more prognosis-oriented feature space. This study suggests that task-specific adaptation is important for medical pretraining.

## Electronic supplementary material

Below is the link to the electronic supplementary material.

Supplementary Material 1

## Data Availability

The LUNG1 dataset is publicly available at the Cancer Imaging Archive (TCIA) repository: https://www.cancerimagingarchive.net/collection/nsclc-radiomics. Other datasets used in this study are available upon reasonable request for academic research purposes from the corresponding author or first author.

## Abbreviations

3D: Three-dimensional
AUC: Area under the receiver operating characteristic curve
CLIP: Contrastive language-image pretraining
CT: Computed tomography
OS: Overall survival
PFS: Progression-free survival
ROC: Receiver operating characteristic
SE: Squeeze-and-excitation
UMAP: Uniform manifold approximation and projection C-index: Concordance index

## Code availability

The source code of this study is publicly available at https://github.com/aaaa222333/ProtoSurv.git.
