# Supplementary material for: ProtoSurv: prototype-guided adaptation of computed tomography foundation model for lung-cancer prognosis prediction
Source: Vis Comput Ind Biomed Art. 2026 Sep 22;9:21. doi: 10.1186/s42492-026-00233-1 (PMC13598028; doi:10.1186/s42492-026-00233-1)
Supplement: Supplementary file 1 [file 42492_2026_233_MOESM1_ESM.pdf]

Supplementary Material

1 Supplementary Methods

1.1 Data Preprocessing

We employed the U-Net model from the `lungmask` library to automatically segment the lung regions from chest CT scans. The resulting 3D lung mask was then used to define a volumetric bounding box that fully enclosed the lung field, creating a lung-centered thoracic region of interest (lung-ROI). This region preserves the entire lung while retaining limited surrounding anatomical structures, such as the pleura and mediastinum. This preprocessing step helps reduce irrelevant anatomical information and standardizes the input, while maintaining clinically significant peri-pulmonary context. The lung-ROI was further processed by clipping voxel intensities to the range [−1500, 500], applying min-max normalization to [0, 1], and resizing the image to a fixed resolution of $48\times256\times256$ voxels.

1.2 Details of Clinical Model

For the clinical model, we evaluated several conventional machine learning classifiers, including support vector machine, logistic regression, random forest, K-nearest neighbor (KNN), Gaussian naive Bayes, decision tree, multilayer perceptron, and gradient boosting. KNN achieved the best validation performance and was used as the final clinical model for both prognosis tasks. For EGFR-TKI PFS prediction, the KNN classifier incorporated five clinical features: age, gender, overall stage, family history of cancer, and smoking history. For (chemo-)radiotherapy OS prediction, the KNN classifier incorporated seven clinical features: age, clinical T stage, clinical N stage, clinical M stage, overall stage, histology, and gender.

1.3 Details of Semi-supervised Baselines

We additionally compared ProtoSurv with one supervised transfer baseline and two semi-supervised learning baselines using the same pretrained feature encoder, labeled training sets, testing sets, and downstream prognostic classifier.

For the supervised transfer baseline, referred to as Supervised transfer, the prognostic classifier was trained directly on the labeled training samples using binary cross-entropy loss, without incorporating any unlabeled samples or pseudo-labeling procedure.

For confidence-based pseudo-labeling, a binary classifier was first trained using the labeled training samples and was then applied to the unlabeled pretraining data. High-confidence predictions were retained as categorical pseudo-labels, and the labeled and pseudo-labeled samples were jointly used to optimize the downstream classifier using cross-entropy loss. This baseline did not use prototype similarity, hierarchical sample assignment, or feature calibration.

For FixMatch, each unlabeled feature was transformed into weakly and strongly augmented views. The weakly augmented feature was used to generate a pseudo-label, and only predictions with a maximum class probability of at least 0.95 were retained. The retained pseudo-labels were then used to supervise the corresponding strongly augmented features. The total objective was the sum of the supervised classification loss and the unsupervised consistency loss, with the unsupervised loss weight set to 1.0.

1.4 Details of the Native Cox-loss Baseline

For the native Cox-loss baseline, the visual encoder was initialized using the same CT-report vision–language pretraining as ProtoSurv. Unlike the fixed-horizon binary prognosis formulation used in the primary experiments, which dichotomized patients according to the median event time, this model used the complete observed survival times and censoring indicators and was directly optimized using the Cox partial likelihood loss. It did not incorporate prototype-guided pseudo-label assignment, confidence filtering, or feature calibration. The same training and testing splits were used, and performance was evaluated using Harrell's C-index, which measures prognostic concordance over the entire follow-up period rather than at a single fixed time point.

1.5 Prototype-guided Pseudo-label Assignment and Feature Calibration

1.5.1 Prognosis-oriented Anchor Classifier Construction

We constructed a prognosis-oriented anchor classifier based on the pretrained visual encoder to establish an initial prognosis-aware decision boundary on limited labeled data. Specifically, for each input sample, the pretrained visual encoder extracts a 400-dimensional feature vector. This feature vector is then fed into a single-layer fully-connected classification head, which directly maps it to binary classification probabilities. The anchor classifier is trained on the labeled dataset using binary cross-entropy loss, during which the parameters of the pretrained encoder are frozen and only the classification head is optimized to prevent overfitting on the limited labeled data. For samples from the large-scale unlabeled dataset, this classifier outputs confidence probabilities for each class, serving as a measure of semantic clarity in the subsequent pseudo-label filtering stage. Only samples satisfying class-specific confidence thresholds are retained as high-quality candidates for the prototype-guided feature calibration stage.

1.5.2 Prototype-guided Pseudo-label Assignment and Feature Calibration

In the HUAXI and LUNG1 datasets, we constructed class prototypes as the centroids of labeled sample features. For HUAXI, class 0 (201 samples) had Q1/Q3 similarities of 0.786/0.917, and class 1 (205 samples) had 0.792/0.927. For LUNG1, class 0 (166 samples) had Q1/Q3 of 0.608/0.816, and class 1 (170 samples) had 0.707/0.855. We defined Q3 and Q1 as τ_{high} and τ_{low}, respectively, for hierarchical pseudo-label assignment. Applying these thresholds to the pretraining dataset, we screened 4,287 candidate samples for HUAXI and 38,886 for LUNG1 for subsequent confidence filtering.

In the LUNG1 dataset, 38,886 initial candidates yielded 34,566 consistent samples. After anchor classifier confidence filtering (thresholds: 0.872 for class 0, 0.922 for class 1), 26,177 high-quality pseudo-labeled samples were retained. In the HUAXI dataset, all 4,287 initial candidates passed consistency verification, and no additional confidence filtering was applied due to generally high confidence scores.

During data loading, feature calibration is performed for weak-consistency samples based on the `need_pull` flag. For each sample requiring calibration, we retrieve the corresponding prototype and thresholds, dynamically compute α ($\eta = 0.75$ for HUAXI, $\eta = 0.10$ for LUNG1; see ablation study), and apply $f' = (1 - \alpha)f + \alpha P_c$. The calibrated feature is then normalized to $[-1, 1]$. Samples without calibration undergo only normalization. This process is implemented via the `preprocess` transformation in `prepare_dataset`.

1.5.3 Prognostic Classifier

For HUAXI and LUNG1, we designed tailored prognostic classifiers. For LUNG1, full ResNet1D maps 400-dim to 512-dim via input projection, followed by three multi-scale stages each with two residual blocks and SE attention, with dimensions $512 \rightarrow 256 \rightarrow 128$; the classification head outputs binary probabilities. For HUAXI, simplified ResNet1D uses 4 residual blocks with SE attention, with dimensions $256 \rightarrow 128 \rightarrow 64$.

2 Supplementary Figures

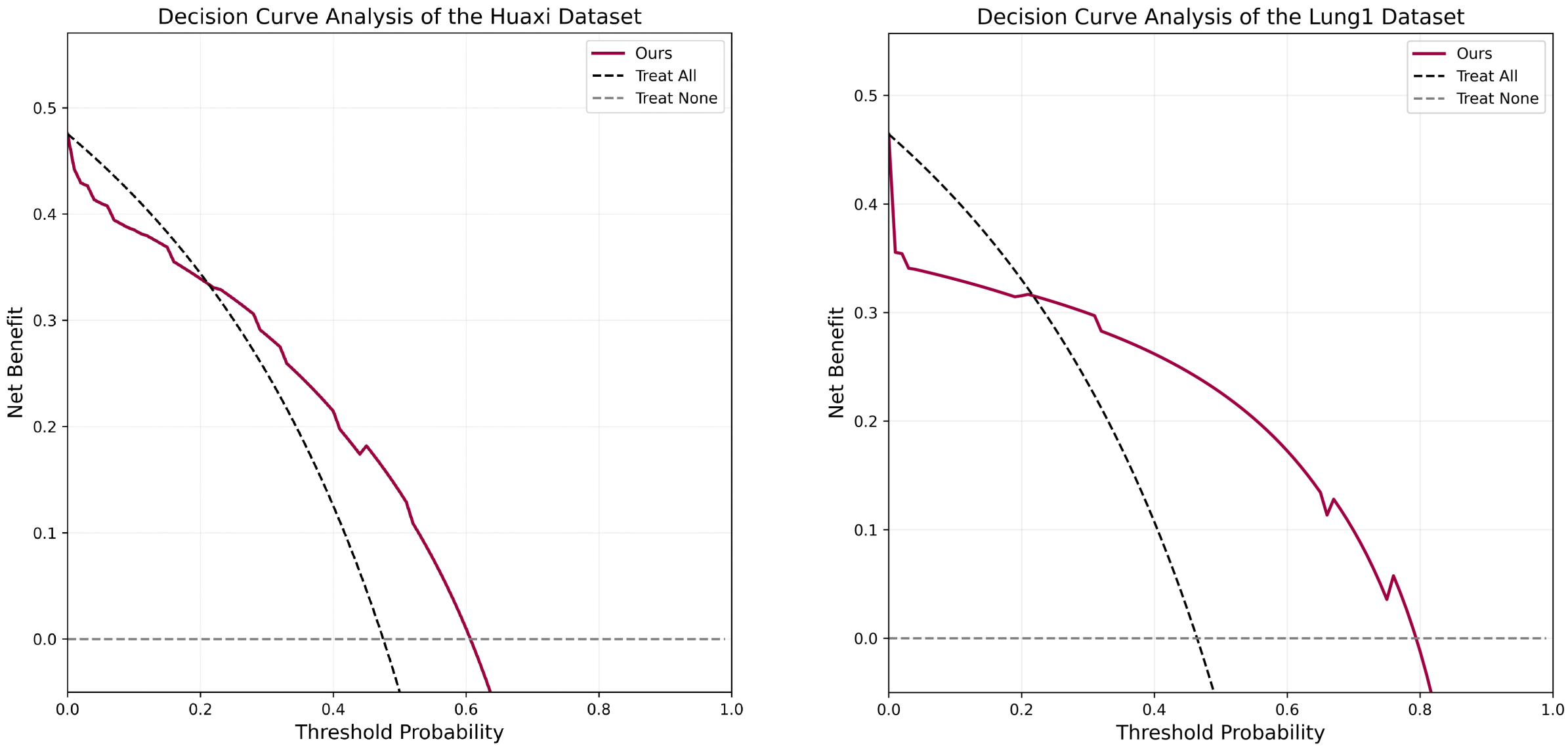

(a) (b)

Fig. S1: Decision curve analysis of ProtoSurv on the HUAXI and LUNG1 datasets. (a) Decision curve analysis on the HUAXI dataset. (b) Decision curve analysis on the LUNG1 dataset.

3 Supplementary Tables

Table S1: Demographic characteristics and CT acquisition parameters of the pretraining cohort.

**Characteristic**	**Value**
**Cohort characteristics**	
Institution	First Hospital of Jilin University
Enrollment period	May 2023–October 2023
Patients, n	104,783
CT sequences, n	409,261
Age, years, mean ± SD	50.2 ± 19.4
Male, n (%)	50,505 (48.2%)
**CT acquisition parameters**	
Scanner manufacturers	Siemens, Philips, GE Healthcare, and United Imaging
Tube voltage, kV	90–120
Tube current, mA	128–594
Reconstruction field of view, mm	223–471
Slice thickness, mm	0.62–8.00
Reconstruction kernels	Multiple vendor-specific soft-tissue and lung kernels

Table S2: Baseline characteristics of patients in the HUAXI and LUNG1 datasets.

Dataset	Characteristic	Training set	Testing set	P value
HUAXI	Age	58.8±10.5	57.5±11.0	0.250
	Sex			0.086
	Female	55.9%	65.3%	
	Male	44.1%	34.7%	
	Overall stage			0.326
	IIIB	3.0%	6.9%	
	IIIC	2.2%	1.1%	
	IVA	54.2%	55.4%	
	IVB	40.6%	36.6%	
	Family history of cancer			0.346
	Yes	19.5%	21.8%	
	No	80.5%	78.2%	
	Smoking history			0.079
	Yes	67.7%	77.2%	
	No	32.3%	22.8%	
LUNG1	Age	68.1±10.2	68.2±9.9	0.935
	Sex			0.456
	Female	33.2%	28.6%	
	Male	66.8%	71.4%	
	Overall stage			0.633
	I	17.5%	17.1%	
	II	9.4%	10.0%	
	IIIA	44.8%	40.0%	
	IIIB	28.3%	32.9%	
	Histology			0.943
	Squamous cell carcinoma	41.3%	40.0%	
	Large cell carcinoma	30.1%	28.6%	
	Adenocarcinoma	12.9%	15.7%	
	Not otherwise specified	15.7%	15.7%	
	T stage			0.917
	T1	17.8%	17.1%	
	T2	39.5%	38.6%	
	T3	12.2%	18.6%	
	T4	30.4%	25.7%	
	N stage			0.927
	N0	37.1%	38.6%	
	N1	5.6%	4.3%	
	N2	36.0%	35.7%	
	N3	21.3%	21.4%	
	M stage			1.000
	M0	100%	100%	

Table S3: Performance comparison of supervised and semi-supervised learning strategies on the HUAXI and LUNG1 datasets.

Dataset	Metric	Supervised transfer	Confidence-based pseudo-labeling	FixMatch	ProtoSurv
HUAXI	AUC	0.7142 [0.6100–0.8144]	0.7551 [0.6577–0.8464]	0.7364 [0.6326–0.8373]	**0.765 [0.673–0.856]**
	Accuracy	0.6238 [0.5347–0.7129]	0.6535 [0.5644–0.7426]	**0.6733 [0.5743–0.7624]**	0.663 [0.574–0.753]
	F1-score	0.6724 [0.5714–0.7612]	0.6847 [0.5862–0.7742]	0.6857 [0.5825–0.7759]	**0.707 [0.614–0.791]**
	Precision	0.5735 [0.4594–0.6912]	0.6032 [0.4833–0.7223]	**0.6316 [0.5094–0.7501]**	0.603 [0.487–0.719]
	Recall	0.8125 [0.7000–0.9231]	0.7917 [0.6739–0.9070]	0.7500 [0.6304–0.8723]	**0.854 [0.755–0.954]**
LUNG1	AUC	0.7932 [0.6916–0.8900]	0.7869 [0.6864–0.8849]	0.7618 [0.6642–0.8578]	**0.822 [0.737–0.903]**
	Accuracy	0.7381 [0.6429–0.8333]	0.7381 [0.6429–0.8217]	0.7024 [0.6068–0.7976]	**0.750 [0.667–0.845]**
	F1-score	0.6857 [0.5429–0.8000]	0.6944 [0.5556–0.8090]	0.6835 [0.5507–0.7879]	**0.734 [0.613–0.832]**
	Precision	**0.7742 [0.6207–0.9032]**	0.7576 [0.5926–0.8929]	0.6750 [0.5135–0.8095]	0.725 [0.576–0.854]
	Recall	0.6154 [0.4571–0.7693]	0.6410 [0.4722–0.7941]	0.6923 [0.5294–0.8333]	**0.744 [0.585–0.881]**

Table S4: Effect of the feature calibration hyperparameter on ProtoSurv performance.

Dataset	Hyperparameter	C-Index	AUC	Accuracy	F1-score
HUAXI	0.00	0.665 [0.596-0.741]	0.738 [0.633-0.935]	**0.683 [0.594-0.772]**	0.667 [0.552-0.771]
	0.05	0.660 [0.594-0.736]	0.731 [0.636-0.827]	0.644 [0.555-0.743]	0.633 [0.517-0.740]
	0.10	0.661 [0.595-0.734]	0.733 [0.631-0.828]	0.654 [0.564-0.743]	0.667 [0.557-0.761]
	0.15	0.661 [0.591-0.737]	0.732 [0.629-0.828]	0.624 [0.535-0.713]	0.628 [0.515-0.726]
	0.20	0.672 [0.605-0.745]	0.748 [0.647-0.844]	0.624 [0.525-0.723]	0.628 [0.512-0.732]
	0.25	0.668 [0.600-0.743]	0.743 [0.640-0.837]	0.644 [0.555-0.733]	0.633 [0.518-0.735]
	0.30	0.677 [0.612-0.754]	0.756 [0.661-0.847]	0.634 [0.545-0.733]	0.648 [0.532-0.748]
	0.35	0.682 [0.620-0.753]	0.762 [0.670-0.849]	0.634 [0.545-0.723]	0.667 [0.569-0.763]
	0.40	0.673 [0.606-0.749]	0.750 [0.651-0.844]	0.644 [0.555-0.743]	0.667 [0.560-0.764]
	0.45	0.679 [0.615-0.752]	0.758 [0.666-0.849]	0.644 [0.555-0.733]	0.654 [0.542-0.755]
	0.50	0.678 [0.613-0.754]	0.757 [0.662-0.848]	0.634 [0.545-0.733]	0.648 [0.535-0.752]
	0.55	0.675 [0.612-0.750]	0.753 [0.660-0.845]	0.634 [0.545-0.723]	0.661 [0.558-0.755]
	0.60	0.670 [0.602-0.747]	0.745 [0.651-0.843]	0.634 [0.545-0.733]	0.654 [0.549-0.752]
	0.65	0.679 [0.616-0.749]	0.758 [0.666-0.852]	0.663 [0.574-0.762]	0.679 [0.575-0.774]
	0.70	0.676 [0.616-0.750]	0.754 [0.662-0.846]	0.663 [0.574-0.753]	0.673 [0.566-0.769]
	0.75	**0.684 [0.622-0.755]**	**0.765 [0.673-0.856]**	0.663 [0.574-0.753]	**0.707 [0.614-0.791]**
	0.80	0.676 [0.613-0.749]	0.754 [0.660-0.847]	0.654 [0.564-0.743]	0.690 [0.593-0.782]
LUNG1	0.00	0.715 [0.650-0.788]	0.804 [0.714-0.888]	0.762 [0.667-0.845]	0.722 [0.582-0.829]
	0.05	0.713 [0.649-0.785]	0.803 [0.706-0.890]	0.750 [0.655-0.833]	0.727 [0.592-0.830]
	0.10	**0.727 [0.665-0.798]**	**0.822 [0.737-0.903]**	0.750 [0.667-0.845]	0.734 [0.613-0.832]
	0.15	0.719 [0.655-0.793]	0.811 [0.721-0.899]	0.762 [0.667-0.845]	0.737 [0.610-0.838]
	0.20	0.715 [0.647-0.787]	0.805 [0.711-0.890]	0.774 [0.679-0.857]	**0.747 [0.620-0.845]**
	0.25	0.711 [0.645-0.787]	0.801 [0.702-0.889]	0.750 [0.655-0.833]	0.720 [0.586-0.825]
	0.30	0.717 [0.652-0.790]	0.808 [0.714-0.892]	0.738 [0.643-0.821]	0.703 [0.563-0.810]
	0.35	0.721 [0.656-0.793]	0.814 [0.723-0.899]	0.738 [0.643-0.822]	0.677 [0.526-0.794]
	0.40	0.718 [0.652-0.789]	0.808 [0.715-0.896]	0.702 [0.607-0.798]	0.615 [0.448-0.735]
	0.45	0.714 [0.644-0.787]	0.805 [0.703-0.896]	0.738 [0.643-0.821]	0.677 [0.528-0.791]
	0.50	0.719 [0.653-0.793]	0.811 [0.721-0.900]	0.774 [0.679-0.857]	**0.747 [0.613-0.846]**
	0.55	0.718 [0.651-0.787]	0.809 [0.716-0.898]	0.714 [0.619-0.798]	0.684 [0.551-0.795]
	0.60	0.720 [0.654-0.790]	0.813 [0.718-0.900]	0.750 [0.655-0.833]	0.720 [0.590-0.822]
	0.65	0.726 [0.665-0.798]	0.821 [0.735-0.902]	0.738 [0.643-0.833]	0.677 [0.528-0.794]
	0.70	0.725 [0.659-0.796]	0.819 [0.729-0.905]	0.738 [0.643-0.821]	0.694 [0.557-0.800]
	0.75	0.721 [0.655-0.794]	0.813 [0.720-0.897]	**0.786 [0.691-0.869]**	0.769 [0.646-0.869]
	0.80	0.719 [0.654-0.791]	0.810 [0.719-0.897]	0.762 [0.678-0.845]	0.744 [0.618-0.842]

Table S5: Additional survival analysis using Cox regression.

Dataset	Model	Hazard ratio	95% CI	P value	Harrell's C-index
HUAXI	ProtoSurv risk score	1.61	1.01–2.57	0.0467	0.638
	Native Cox-loss model	1.004	1.002–1.007	0.0016	0.600
LUNG1	ProtoSurv risk score	2.20	1.34–3.61	0.0018	0.680
	Native Cox-loss model	1.007	1.003–1.011	0.0005	0.652
